# Liquid Chromatography-Tandem Mass Spectrometry Simultaneous Determination and Pharmacokinetic Study of Fourteen Alkaloid Components in Dog Plasma after Oral Administration of *Corydalis bungeana Turcz* Extract

**DOI:** 10.3390/molecules23081927

**Published:** 2018-08-02

**Authors:** Hongrui Dong, Guanyun Yan, Zhibin Wang, Chengcui Wu, Binbin Cui, Yixuan Ren, Chunjuan Yang

**Affiliations:** 1 Department of Pharmaceutical Analysis and Analytical Chemistry, College of Pharmacy, Harbin Medical University, No. 157 Baojian Road, Nangang District, Harbin 150081, Heilongjiang, China; donghongrui422@163.com (H.D.); 18845645250@163.com (C.W.); binbincui0419@163.com (B.C.); renyixuan1218@163.com (Y.R.); 2 Department of Pharmacy Management Harbin Medical University, Harbin 150086, Heilongjiang, China; hydygy@hrbmu.edu.cn; 3 Key Laboratory of Chinese Materia Medical (Ministry of Education), Heilongjiang University of Chinese Medicine, Harbin 150040, Heilongjiang, China; wzbmailbox@hljucm.net

**Keywords:** Ultra high-performance liquid chromatography-tandem mass spectrometry, *Corydalis bungeana Turcz*, alkaloids, pharmacokinetics

## Abstract

A rapid and sensitive Ultra high-performance liquid chromatography-tandem mass spectrometry (UHPLC-MS/MS) method was developed for the simultaneous determination of fourteen alkaloids in beagle dog plasma after a single oral dose of the *Corydalis bungeana Turcz* (*C. bungeana*) extract selected bifendate as the internal standard (IS). The plasma samples were preprocessed by liquid-liquid extraction (LLE) with aether before separation on an Agilent SB-C_18_ column (1.8 µm, 150 × 2.1 mm) using a gradient elution program. The mobile phase consists of 0.2% acetic acid and acetonitrile at the flow rate of 0.3 mL/min. In the positive ion mode, the analytes were detected by multiple reaction monitoring (MRM). The results indicated that calibration curves for fourteen analytes have good linearity (*R*^2^ = 0.9904). The lower limits of quantification (LLOQ) of fourteen alkaloids and IS were all over 4.87 ng/mL and the matrix effects ranged from 94.08% to 102.76%. The mean extraction recoveries of Quality control samples at low (LQC), medium (MQC) and high (HQC) and IS were all more than 78.03%. The intra- and inter-day precision (R.S.D.%) also met the criterion, at the same time the deviation of assay accuracies (R.E) ranged from −13.70% to 14.40%. The *T*_max_ values of fourteen alkaloids were no more than 1 h. The range of *C*_max_ was from 74.16 ± 8.71 to 2256 ± 255.9 ng/mL. The assay was validated in the light of the regulatory bioanalytical guidelines and proved acceptable, which was successfully applied to a pharmacokinetic study of these compounds in beagle dogs after oral administration of *Corydalis bungeana Turcz* extract.

## 1. Introduction

*Corydalis bungeana Turcz*. (*C. bungeana*), also known as “Diding”, “Kuding” or “Xiaojicai” and first seen in the “Annals of traditional Chinese Medicine,” belongs to the Papaveraceae family. The dried whole plant is referred in traditional Chinese medicine (TCM) as *Herba Corydalis Bungeanae* and is officially listed in Chinese Pharmacopoeia [1]. It is a perennial herb with violet to pink flowers distributed in the northern and eastern parts of China, the southeast of Mongolia, the northern part of the Korean peninsula and the far east of Russia.

The botanical medicine is used for clearing away heat and toxic materials, activating blood circulation and detumescence, treating various kinds of inflammation such as cold and cough, especially for rheumatism and myocarditis [2]. Furthermore, *C. bungeana* has such a good anti-inflammatory effect due to restrain the activation of NF-κB signaling pathway through inhibiting phosphorylation of IκBα and p65. In addition, *C. bungeana* attenuates inflammatory reaction by controlling the expression of various inflammatory cytokines both *in vivo* and *in vitro* [3]. *C. bungeana* clinically was applied in many traditional formulas, such as Ganmaoqingre Granules and Cholagogic Eight Flavor Scattered, Clearing Blood Detoxification Pill, Forsythia Detoxifacational Pill (powder) and so forth. 

*C. bungeana* possesses abundant alkaloids, containing 7′-(3′,4′-dihydroxyphenyl)-*N*-[(4-me thoxyphenyl)-ethyl]propena-mide (A/Z23), coptisine (B), berberrubine (C), sanguinarine (D), worenine (E), berberine (F), jateorhizine (G), columbamine (H), palmatine (I), protopine (J), tetrahydropalmatine (K), corynoline (L), 8-oxocorynoline (M), acetylcorynoline (N) and so on. Besides, alkaloids were the major ingredients and numerous scientific pharmacological studies showed above fourteen compounds and extracts containing them had the common significant anti-inflammatory and antibacterial effects. Corynoline, the only ingredient to assess the quality control of this herbal medicine in the Chinese Pharmacopoeia [4], has been found that it also has therapeutic effect on LPS-induced ALI (Acute lung injury) in mice by restraining inflammatory response [5]. In addition, corynoline, acetylcorynoline and protopine not only can significantly reduce carbon tetrachloride (CCl4)- induced microsomal lipid peroxidation [6] and attenuate two, 4-dinitro-1-fluorobenzene-induced delayed-type hypersensitivity [5] but also can protect against experimental liver injury [7]. Pharmacological study also suggested that protopine and palmatine has anti-parasitic [8] and anti-arrhythmic [9] effects. Furthermore, it is demonstrated that palmatine can induce cell apoptosis to kill breast cancer cells MCF-7 [10] and tetrahydropalmatine exhibits a substantial protective action against ischemic injury in neuronal and cardiac tissues [11,12]. Columbamine, a safe natural compound, has been reported to possess anti-hyperglycemic, anti-nociceptive, antiplasmodial, lipoxygenase-inhibiting and anti-oxidant effects [13,14,15]; Coptisine, berberine and sanguinarine has the anti-cancer activity [16]. Furthermore, the anticancer effects of sanguinarine act on many cancers, including neuroendocrine, sarcoma, osteosarcoma and human neuroblastoma cells [17,18,19,20]. Interestingly, it is reported that coptisine can inhibited proliferation of vascular smooth muscle cells (VSMCs) at a lower concentration [21]. So, coptisine may be a new active ingredient curing atherosclerosis (AS). Meanwhile, berberine, the homologue of jateorhizine and the most studied alkaloid in Coptis chinensis, which can improve hyperlipidemia and insulin resistance [22,23]. Berberrubine showed good antibiosis, anti-oxidant effects on human fibrosarcoma cells and the scavenging effect of reactive oxidative species (ROS) [24,25]. 

In our previous investigations, quantitative analysis of alkaloids in *C. bungeana* have been reported in the literature of thin optical density scanning method, high performance liquid chromatography including reversed-phase HPLC method and liquid chromatography with tandem mass spectrometry method (LC-MS/MS) [26,27,28,29]. It is reported that only two publications have developed LC-MS/MS method, which measured 2 alkaloids (corynoline, acetylcorynoline) and 5 alkaloids (protopine, corynoline, Z23, acetylcorynoline and 8-oxocorynoline), respectively. [30]. As far as we known, no published methods have been reported for the simultaneous determination of fourteen alkaloids of *C. bungeana* extraction in biological samples (both animal and human body).

In this paper, we aimed to develop a rapid, sensitive and specific LC-MS/MS method for the quantification of fourteen alkaloids of *C. bungeana* in Beagle dog plasma. And the developed method was supposed to be applied to a pharmacokinetic study of fourteen alkaloids after oral administration of extract of *C. bungeana* in Beagle dog plasma.

## 2. Results and Discussion

### 2.1. UHPLC-MS/MS Optimization

The full-scan mass spectra revealed that the ionizations of the fourteen alkaloids were adequately high response in the positive-ion mode for detection. Due to the complexity of the detection method, the MS/MS ion transitions were taken in multiple reaction monitoring (MRM) mode, which can improve the specificity of the detection method. To gain the richest relative abundance of the precursor and product ions, some parameters such as fragmentor, collision energy, capillary voltage and source or desolvation temperature were optimized. The fragmentors were at 180 V for acetylcorynoline, 170 V for protopine and 8-oxocorynoline, 140 V for Z23, 147 V for coptisine, 160 V for berberrubine, corynoline and columbamine, 171 V for sanguinarine, 181 V for worenine, 136 V for berberine, 148 V for jateorhizine, 158 V for palmatine, 159 V for tetrahydropalmatine. The collision energy (CE) were set at 25 V for corynoline and 8-oxocorynoline and 50 V for acetylcorynoline and 30 V for protopine, palmatine and berberine and 20 V for Z23 and 29 V for coptisine, berberrubine and columbamine and 35 V for sanguinarine and 48 V for worenine and 28 V for jateorhizine and 27 V for tetrahydropalmatine, respectively. The other MS parameters were adopted according to the recommended values for the instrument. 

Chromatographic conditions were investigated to achieve excellent separations, observe desired peak shapes, improve signal responses and appropriate run time. The mobile phase played a vital part in this process. In order to obtain optimum elution conditions, we tried several mobile phases including methanol-water, acetonitrile-water, acetonitrile-acetic acid and methanol-ammonium acetate systems. The results showed that the height of the chromatographic peak using methanol-water and methanol-ammonium acetate systems is extremely low. And peak shape of chromatograph of acetonitrile-water was rather unsatisfactory. Ultimately, acetonitrile-acetic acid provided better peak shapes and a more satisfactory baseline than the other mobile phase compositions. The addition of acetic acid into the mobile phase could improve the sensitivity and separation efficiency. To achieve the best solvent system for elution, different proportions of acetic acid (0.1%, 0.2% and 0.3%) were tried. The best response was obtained from 0.2% acetic acid in water (A) and acetonitrile (B) within lower background noise. Different columns including a 5 cm C_18_ column and a 15 cm C_18_ column were tested for optimal condition. It was found that an Agilent SB-C_18_ column (1.8 µm, 150 × 2.1 mm) provided approving resolution, peak symmetry and retention time. The analytes were eluted via a gradient elution at a flow rate of 0.3 mL/min and the column oven temperature was fixed to 40 °C. Fourteen analytes were separated smoothly in 6.62 min without interference from other ingredients in beagle dog plasma. 

### 2.2. Selection of Extraction Method

The sample treatment method is significant for improving extraction recovery and reducing matrix effect. Protein precipitation, liquid-liquid extraction are major methods of plasma treatment in general. In the study, we first used methanol, acetonitrile and methanol-acetonitrile (1:1/1:2, *v*/*v*) to precipitate the protein, the results illustrated that the samples had severe matrix effect and low extraction recovery. Furthermore, the stability of the samples is so negative that the reproducibility down to the below grade. Alternatively, ethyl acetate, cyclohexane, aether and dichloromethane was tested by liquid-liquid extraction method and aether was adopted.

### 2.3. Selection of IS

When performing the pharmacokinetic study, it is important to select the suitable IS. The IS is not supposed to react with the analytes and should not interfere with the determination of target compounds, which is the chief gauge for the selection of internal standards. Furthermore, internal standard will affect the variability in extraction, UHPLC injection and ionization. Several compounds such as phenacetin, theophylline and bifendate were being tested as the IS. However, the polarity of some alkaloids such as corynoline and acetylcorynoline were too low for them to be eluted under the selected UHPLC condition with phenacetin. The peak shape of theophylline was unsatisfactory. Meanwhile, the preliminary experiment found that the bifendate is not a component in *C. bungeana* and exhibited a good response, peak shape and no significant direct interference. Thus, bifendate is more suitable for measuring the concentration of fourteen alkaloids. 

### 2.4. Method Validation

#### 2.4.1. Selectivity 

**Figure 1:**
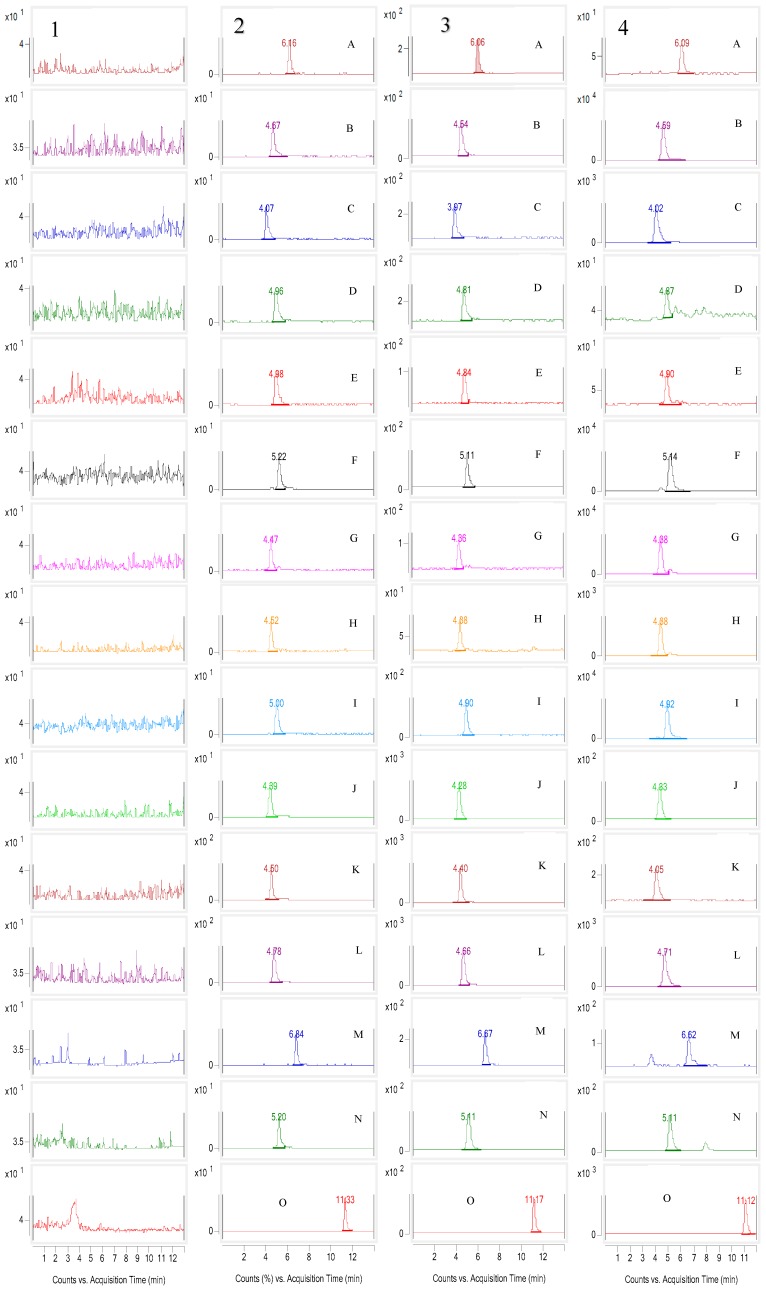
(1) Blank sample (without the fourteen analytes and I.S.) (2) Blank plasma spiked with the fourteen analytes and I.S. (LLOQ: 5.34/5.00/5.53/5.00/5.55/5.40/5.00/5.58/5.65/4.87/5.07/5.11/5.80/6.50 ng/mL for Z23 (**A**), coptisine (**B**), berberrubine (**C**), sanguinarine (**D**), worenine (**E**), berberine (**F**), jateorhizine (**G**), columbamine (**H**), palmatine (**I**), protopine (**J**), tetrahydropalmatine (**K**), corynoline (**L**), 8-oxocorynoline (**M**), acetylcorynoline (**N**)). (3) Blank plasma spiked with fourteen alkaloids and I.S (QCM: 133.5/125.0/138.1/125.0/138.8/135.0/125.0/139.4/141.3/121.8/126.8/127.8/145.0/162.5 ng/mL for **A**, **B**, **C**, **D**, **E**, **F**, **G**, **H**, **I**, **J**, **K**, **L**, **M**, **N**). (4) Representative MRM chromatograms of (**A**–**N**) and IS in a beagle dog plasma taken 1 h after administration of 0.12 g/kg *C. bungeana* extract.

The selectivity of the method towards endogenous plasma matrix was assessed by plasma from six beagle dogs. Figure 1 shows the representative MRM chromatograms of the blank beagle dog plasma (1), blank beagle dog plasma spiked with the analytes at LLOQ (2) and QCM (3) and an *in vivo* plasma sample obtained at 1h after oral administration of *C. bungeana* extract (4). The retention time were about 6.09, 4.59, 4.02, 4.87, 4.90, 5.14, 4.38, 4.38, 4.92, 4.33, 4.05, 4.71, 6.62, 5.11 for A–N, respectively. No co-eluting peaks or interfering signals were observed at the retention time of the analytes and IS.

#### 2.4.2. Linearity and LLOQ

The regression equations, correlation coefficients and linearity ranges for fourteen analytes are summarized in Table 1. All calibration curves exhibited good linearity with correlation coefficients within the confines of 0.9904–0.9989. The lower limits of quantification (LLOQ) were 5.34, 5.00, 5.53, 5.00, 5.55, 5.40, 5.00, 5.58, 5.65, 4.87, 5.07, 5.11, 5.80, 6.50 ng/mL for compounds A–N, respectively, which were applied sufficiently to pharmacokinetic studies.

#### 2.4.3. Accuracy and Precision

Intra- and inter-day precision and accuracy of the method were evaluated by measuring six replicates of the QC samples at three concentration level (LQC, MQC and HQC) and LLOQ and the complete analytical runs was carried on the same day for three consecutive days (Table 2). The accuracy data in the present study ranged from −13.70% to 14.40% (RE) and the intra- and inter-day precision (RSD) is shown in Table 2, respectively. The results state clearly that the method was reliable for the determination for beagle dog plasma samples. 

#### 2.4.4. Extraction Recovery and Matrix Effect 

The extraction recovery and matrix effect of fourteen analytes were investigated by analyzing QC samples at the three evaluated concentrations including low, medium and high with six replicates. Table 3 summarizes the extraction recovery and matrix effect of fourteen analytes and IS. The mean recoveries of the fourteen analytes were in the range of 78.03–89.67% and the mean recovery of the IS was 84.71 ± 8.97%. The corresponding matrix effect derived from QC sample were between 94.08 and 102.76 and it was 99.12 ± 11.88 for the IS. The results displayed that there was no significant ion suppression or enhancement from plasma for this assay.

#### 2.4.5. Stability

As illustrated in Table 4. The stability of QC samples at three evaluated concentrations contained low, medium and high under different stored conditions was assessed on the basis of peak areas in comparison with freshly prepared QC sample. The results of stability assay indicated that these analytes were all stable with accuracy (expressed as RE) in the range from −8.16% to 13.95%.

### 2.5. Pharmacokinetic Study

**Figure 2:**
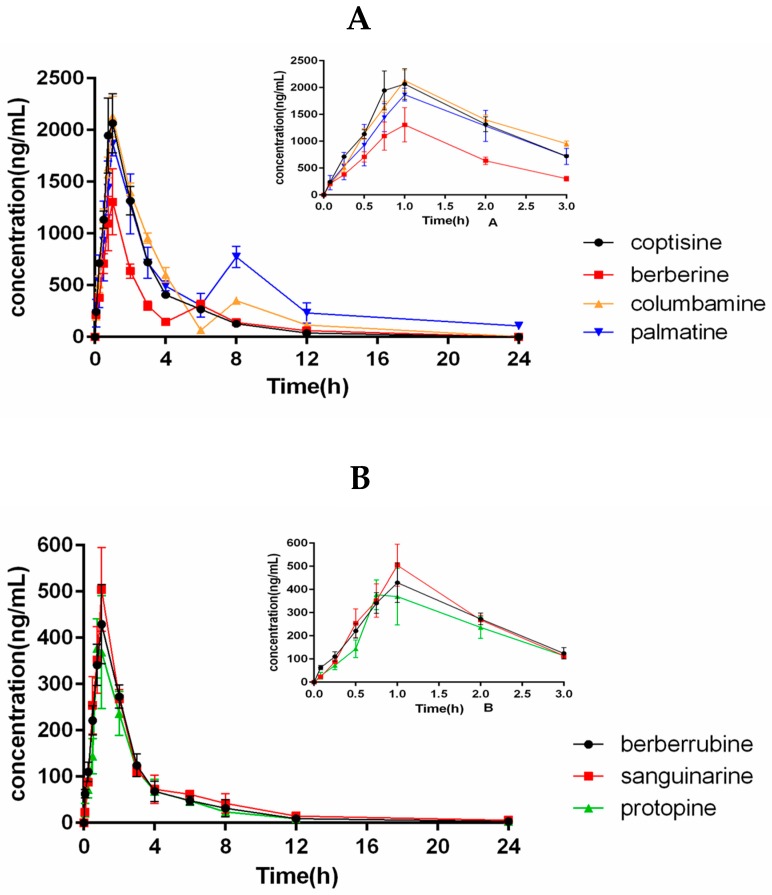

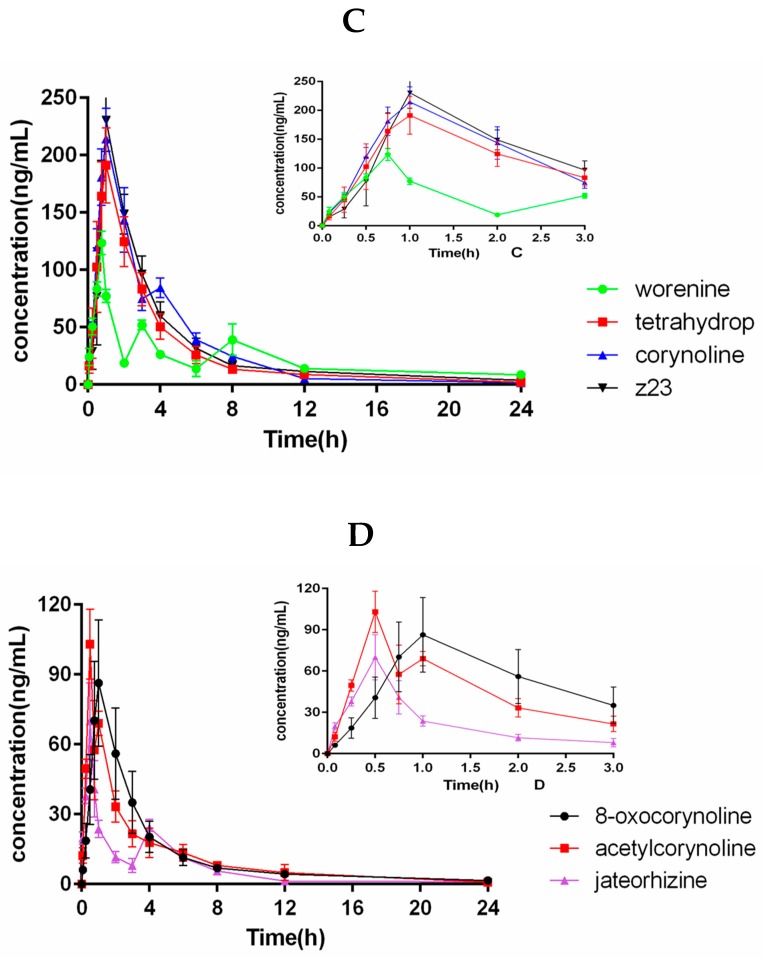
The concentration of fourteen alkaloids in plasma-time profiles of Z23, coptisine, berberrubine, Sanguinarine, worenine, berberine, jateorhizine, columbamine, Palmatine, protopine, tetrahydropalmatine, corynoline, 8-oxocorynoline, acetylcorynoline in beagle plasma after oral administration of *C. bungeana* extract. Values are expressed as mean ± SD (*n* = 6). The mean ± SD plasma concentration-time profiles of the fourteen alkaloids in the beagle dog plasma after oral administration of *C. bungeana* extract (**A**–**D**) from 0 to 3 h, respectively. (*n* = 6).

The developed UHPLC-MS/MS method was employed to determine the pharmacokinetics of the fourteen components in *C. bungeana* after a single oral administration of 0.12 g/kg (equivalent to 1180.8 µg/kg A, 100.8 µg/kg B, 172.8 µg/kg C, 194.4 µg/kg D, 147.6 µg/kg E, 291.6 µg/kg F, 7.2 µg/kg G, 64.8 µg/kg H, 10.8 µg/kg I, 435.6 µg/kg J, 7.2 µg/kg K, 2851.2 µg/kg L, 694.8 µg/kg M, 928.8µg/kg N) to six beagle dogs. We have chosen an oral dosage equivalent to the human dosage prescribed by the Chinese Pharmacopoeia for dogs (2015 edition). The Chinese Pharmacopoeia specifies that the human dosage of *C. bungeana* is 15 g/day. According to the Meeh-Rubn method, we established a final oral dosage of 0.12 g/kg in dogs. The plasma concentrations of the fourteen components at each time point were detected and the data gained from six beagle dogs were averaged. The plasma concentration-time profiles of compounds A–N are shown in Figure 2 with corresponding pharmacokinetic parameters calculated using non-compartmental model showed in Table 5.

The *T*_max_ values of A–N were achieved in 0.96, 0.92, 0.96, 0.96, 0.71, 0.92, 0.54, 0.92, 0.96, 0.88, 0.92, 0.96, 0.92, 0.58 h, respectively. All analytes attained their maximum concentration in beagle dog plasma about 1 h after oral administration, which indicated that the entrance of these ingredients *in vivo* was through absorption in the stomach. Our group investigated the pharmacokinetics of the Z23, protopine, corynoline, 8-oxocorynoline and acetycorynoline in *C. bungeana* extract after a single oral administration to rats in the past. The *T*_max_ value of Z23 and protopine were achieved in 3.71 h and 1.96 h in the rat plasma [30]. A comparison of the two experiments indicates that Z23 and protopine are absorbed faster in beagle dog plasma than in rat plasma. This phenomenon may be due to the differences among species. The *C*_max_ values are 237.0 ± 13.66, 2256 ± 255.9, 450.1 ± 61.61, 533.4 ± 29.12, 123.4 ± 10.34, 1370 ± 312.7, 74.16 ± 8.71, 2130 ± 195.7, 1902 ± 84.86, 433.7 ± 73.92, 205.0 ± 22.39, 223.9 ± 15.29, 94.49 ± 22.77, 106.7 ± 4.630 ng/mL for A–N, respectively. These results may be due to the differences in the contents of the fourteen compounds in the *C. bungeana* extract. Furthermore, Lau et al. [31] reported that many herbs and natural compounds isolated from herbs have been identified as substrates, inhibitors and inducers of various CYP3A4 and herb-CYP interactions may occur and affect the pharmacokinetics. The range of AUC_0–t_ was from 151.1 ± 6.420 to 8037 ± 459.3 ng h/mL. The range of AUC_0–∞_ was from 163.2 ± 11.04 to 9582 ± 596.2 ng h/mL. The half-life of elimination (*t*_1/2_) ranged from 2.48 ± 0.33 to 8.56 ± 1.29 h, which suggests that the fourteen components were rapidly eliminated in beagle dog plasma after oral administration of *C. bungeana* extract. It also showed conspicuous bimodal phenomenon in plasma concentration-time profiles of berberine, jateorhizine, columbamine, palmatine and corynoline. The first peak appeared at 0.5–1 h and the second peak occurred at 4–8 h post-dose. The concentration of palmatine retained at quite a high level for several hours, while jateorhizine showed a faster elimination process. Triple peaks were appeared in curves of mean plasma concentration for worenine. Deng et al. [32] observed multiple blood concentration peaks in alkaloid pharmacokinetics, probably due to distribution re-absorption and enterohepatic circulation. The comprehensive investigations provided important information for evaluating pharmacokinetics, pharmacy and toxicity of the TCM *C. bungeana*, which is helpful for promoting research on its efficacy in clinical therapeutic studies.

## 3. Materials and Methods 

### 3.1. Material and Reagents

Reference standards of coptisine (140,430), berberrubine (140,407), worenine (150,524), berberine (141,128), columbamine (141,108), palmatine (140,821) and tetrahydropalmatine (151,123) with over 98% purity were purchased from the Chengdu pufei De Biotech Co. Ltd. (Chengdu, Sichuan province, China). Sanguinarine (MUST-14082303), jateorhizine (MUST-16110702), protopine (MUST-12030101), corynoline (MUST-16092710) and acetylcorynoline (MUST-14032003) with over 98% purity were purchased from the Chengdu MUST Bio-technology Co. Ltd. (Chengdu, Sichuan province, China). Z23 and 8-oxocorynoline with 98% purity, determined on the basis of UV, MS, NMR and HPLC analysis, were isolated from *C. bungeana* provided by Harbin Medical University (Harbin, Heilongjiang, China). Bifendate was purchased from the National Institutes for Food and Drug Control (100121-199903, Beijing, China) and used as an internal standard (IS). 

HPLC-grade methanol and acetonitrile were purchased from Amethyst Chemicals (Beijing, China). HPLC-grade acetic acid was purchased from CNW Technologies (Shanghai, China). All the other reagents were of analytical-grade. Ultrapure water was prepared by using a MilliQ water purification system (Millipore, Molsheim, France). Plasma samples were prepared from the blood of beagle dogs.

*C. bungeana* was collected from the Anguo Traditional Chinese herbal medicine Market of Hebei province, in June 2015 and was identified by Professor Lianjie Su of Heilongjiang University of Chinese Medicine.

### 3.2. Instruments and Analytical Conditions

Chromatographic analysis was performed on a UHPLC-MS/MS system (Agilent Technologies 1290 series, (Agilent Technologies, Santa Clara, CA, USA), consisting of a quaternary pump, an automatic degasser and an auto-sampler, coupled to a 6430 QQQ-MS instrument (Agilent, Santa, Clara, CA, USA) with an ESI interface. An Agilent SB-C_18_ column (1.8 µm, 150 × 2.1 mm) was employed in the chromatographic separation. The autosampler injection volume was 10 µL. The outlet column pressure was set at 400 bar and the pressure limit of the system was 1200 bar under these chromatographic conditions. The UHPLC mobile phase was comprised of A (0.2% acetic acid solution) and B (acetonitrile) using a gradient system. The eluent gradient was as follows: 0–1.8 min 28% B (72:28, *v*/*v*); 1.8–3.5 min linear increase to 65% B (35:65, *v*/*v*); 3.5–4.0 min linear increase to 75% B (25:75, *v/v*) at a flow rate of 0.3 mL/min. Lastly, the gradient decreased to 28% B (72:28, *v*/*v*) and the column was equilibrated for 2.5 min before injecting the next sample.

**Figure 3:**
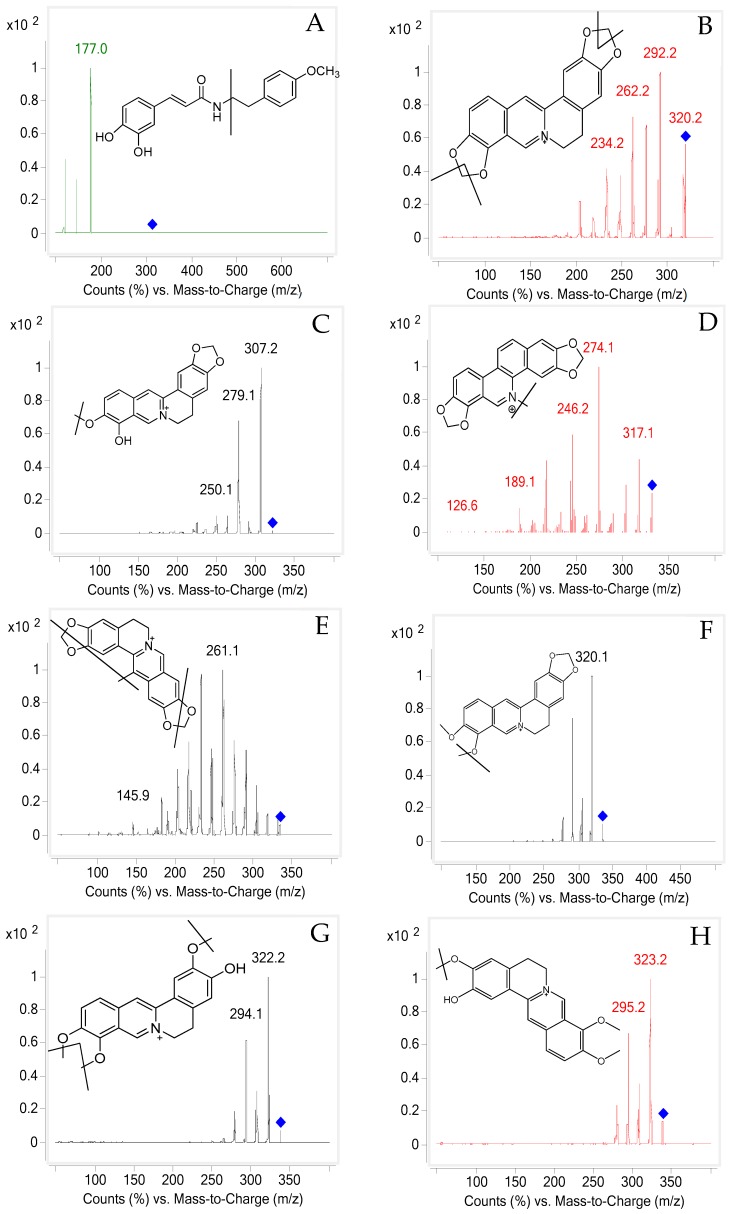

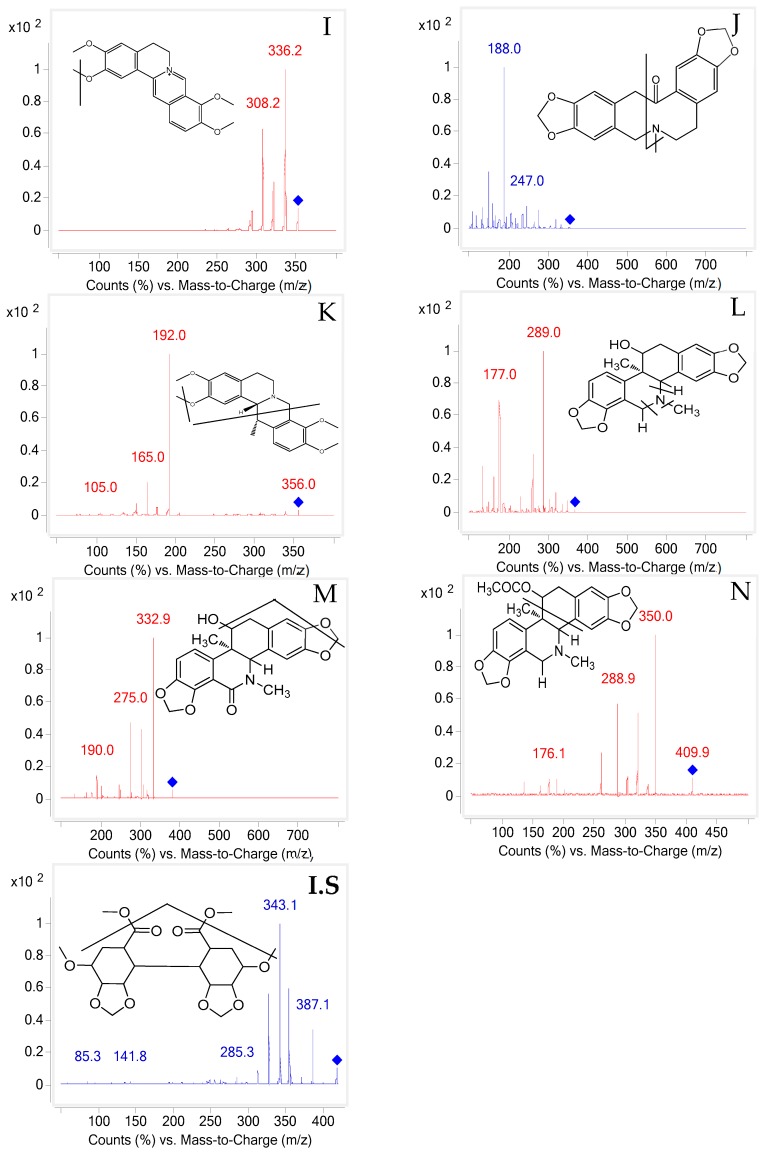
MS/MS fragmentation patterns of (**A**) 7′-(3′,4′-dihydroxyphenyl)-*N*-[(4-me thoxyphenyl)-ethyl]propena-mide/Z23, (**B**) coptisine, (**C**) berberrubine, (**D**) berberrubine, (**E**) worenine, (**F**) berberine, (**G**) jateorhizine, (**H**) columbamine, (**I**) palmatine, (**J**) protopine, (**K**) tetrahydropalmatine, (**L**) corynoline, (**M**) 8-oxocorynoline, (**N**) acetylcorynoline and (**I.S**).

Mass spectrometry detection was performed using an Agilent Mass Hunter workstation. The positive ionization mode was employed for compound ionization. The quantification was obtained in multiple reaction monitoring (MRM) mode with the precursor and product ion transitions at *m*/*z* 314.1→177.0 for A, *m*/*z* 320.2→292.2 for B, 322.2→307.2 for C, *m*/*z* 332.1→274.1 for D, *m*/*z* 334.2→261.1 for E, *m*/*z* 336.2→320.1 for F, *m*/*z* 338.2→294.1 for G, 339.2→323.2 for H, *m*/*z* 352.2→336.2 for I, *m*/*z* 354.1→188.0 for J, *m*/*z* 356.0→192.0 for K, *m*/*z* 368.1→289.0 for L, *m*/*z* 382.1→332.9 for M and *m*/*z* 409.9→350.0 for N, respectively (Figure 3). Selecting high-purity N_2_ as the nebulizing gas and N_2_ plays a role of drying gas at a flow rate of 11 L/min. The parameters in the source were set as follows: capillary voltage 4500 V; source temperature 100 °C; desolvation temperature 350 °C.

### 3.3. Preparation of Corydalis bungeana Turcz Extract

An organic solvent reflux extraction method was employed for the extract preparation. Dried powder (200 g) of *C. bungeana* was extracted three times (1.5 h each time) with 75% ethanol (1:10, *w*/*v*) and then filtered. The combined filtrate was evaporated to dryness and the residue was redissolved in water to achieve a concentration equivalent to 1.35 g/mL of *C. bungeana*. To calculate the administered dose, the contents of fourteen alkaloids in the administration solution were quantitatively determined by LC-MS/MS. The contents of A–N were 13.28 mg/mL, 1.13 mg/mL, 1.94 mg/mL, 2.19 mg/mL, 1.66 mg/mL, 3.28 mg/mL, 0.08 mg/mL, 0.73 mg/mL, 0.12 mg/mL, 4.90 mg/mL, 0.08 mg/mL, 32.08 mg/mL, 7.82 mg/mL, 10.45 mg/mL, respectively.

### 3.4. Preparation of Calibration Standards and Quality Control (QC) Samples

Standard mixed stock solutions of A–N were prepared in methanol at 267.0, 250.0, 276.3, 250.0, 277.5, 270.0, 250, 278.8, 282.5, 243.5, 253.5, 255.5, 290.0, 325.0 μg/mL, respectively and then further diluted in methanol to gain the working solutions with a series of concentrations. The IS solution (2000 ng/mL) was prepared by diluting the stock solution in methanol for routine use. The samples for the standard calibration curves were prepared by spiking proper amounts of the standard solutions and blank plasma to obtain the final concentrations of 5.34, 10.86, 26.70, 133.5, 267.0, 534.0, 2670 ng/mL for Z23 (A); 5.00, 10.00, 25.00, 125.0, 250.0, 500.0, 2500 ng/mL for coptisine (B); 5.53, 11.05, 27.63, 138.1, 276.3, 552.5, 2763 ng/mL for berberrubine (C); 5.00, 10.00, 25.00, 125.0, 250.0, 500.0, 2500 ng/mL for sanguinarine (D); 5.55, 11.10, 27.75, 138.8, 277.5, 555.0, 2775 ng/mL for worenine (E); 5.40, 10.80, 27.00, 135.0, 270.0, 540.0, 2700 ng/mL for berberine (F); 5.00, 10.00, 25.00, 125.0, 250.0, 500.0, 2500 ng/mL for jateorhizine (G); 5.58, 11.15, 27.88, 139.4, 278.8, 557.5, 2788 ng/mL for columbamine (H); 5.65, 11.30, 28.25, 141.3, 282.5, 565.0, 2825 ng/mL for palmatine (I); 4.87, 9.74, 24.35, 121.8, 243.5, 487.0, 2435 ng/mL for protopine (J); 5.07, 10.14, 25.35, 126.8, 253.5, 507.0, 2535 ng/mL for tetrahydropalmatine (K); 5.11, 10.22, 25.55, 127.8, 255.5, 511.0, 2555 ng/mL for corynoline (L); 5.80, 11.60, 29.00, 145.0, 290.0, 580.0, 2900 ng/mL for 8-oxocorynoline (M); 6.50, 13.00, 32.50, 162.5, 325.0, 650.0, 3250 ng/mL for acetylcorynoline (N). The QC samples were prepared in drug-free plasma at four different concentration levels, high QC (2136/2000/2210/2000/2220/2160/2000/2230/2260/1948/2028/2044/2320/2600 ng/mL), medium QC (133.5/125.0/138.1/125.0/138.8/135.0/125.0/139.4/141.3 /121.8/126.8/127.8/145.0/162.5 ng/mL), low QC (10.86/10.00/11.05/10.00/11.10/10.80/10.00/11.15/11.30/9.74/10.14/10.22/11.60/13.00 ng/mL) and LLOQ (5.34/5.00/5.53/5.00/5.55/5.40/5.00/5.58/5.65/4.87/5.07 /5.11/5.80/6.50 ng/mL) for Z23 (A), coptisine (B), berberrubine (C), sanguinarine (D), worenine (E), berberine (F), jateorhizine (G), columbamine (H), palmatine (I), protopine (J), tetrahydropalmatine (K), corynoline (L), 8-oxocorynoline (M), acetylcorynoline (N). In order to ensure the accuracy of the experiment, the calibration standards and the QC samples were abstracted with aether and prepared under the same conditions as the test samples. All stock and preparing working solutions were placed in the freezer at 4 °C until use. 

### 3.5. Animal Experiments

Six healthy male beagle dogs (body weight 10 ± 2 kg) were provided by Shen-yang Kangping Institute of Laboratory Animal (SCXK (Liao) 2014-0003). The animal handling procedures were ratified by the Institutional Ethics Committee and accord with the principles of the International Guide for the Care and Use of Laboratory Animals. Before the experiment, the beagle dogs were fasted for 12 h but given water during the research period. Blood (300 µL) was collected from the forelimb vein plexus at a few specific time points (0.083, 0.25, 0.5, 0.75 and 1, 2, 3, 4, 6, 8, 12, 24 h) after the oral administration of the extract of *C. bungeana* (0.12 g/kg). After centrifugation at 13,000 rpm for 10 min, the plasma was collected immediately and maintained at −80 °C until sample analysis.

### 3.6. Preparation of Plasma Samples

Using a liquid-liquid extraction method, 100 µL of methanol and 50 µL of IS (bifendate) were added to a beagle dog plasma sample (100 µL) followed by vortex for 30 s. Then 3 mL of aether was accurately added and the mixture was vortexed for 60 s. After this step, the samples were put into a centrifuge (XiangYi Technologies, Changsha, Hunan, China). The organic supernatant was pipetted into clean glass tubes individually after centrifugation 3800 rpm for 6 min. The upper organic layer was removed and evaporated to dryness at 40 °C under a stream of nitrogen. The residue was then redissolved with 100 µL of mobile phase (28% acetonitrile), vortexed for 30 s and filtered by a 0.22 µm membrane. A 10 µL aliquot of the solution was injected into the UHPLC-MS/MS system for analysis.

### 3.7. Method Validation 

#### 3.7.1. Specificity 

The specificity was evaluated by comparing the chromatograms of six individual blank plasma with the corresponding plasma samples spiked with the fourteen alkaloids and IS, as well as plasma samples collected after oral administration of extract of *C. bungeana*.

#### 3.7.2. Linearity and Lower Limits of Quantification (LLOQ)

The calibration curve was established by analyzing a series of standard plasma samples at seven different concentrations. The linearity of each calibration curve was determined by plotting the peak area ratio (*y*) of the analyte to the IS against the nominal concentration (*x*) of the analyte with weighted (1/*x*^2^) least square linear regression. The LLOQ was defined as the lowest concentration point of the calibration curve at which can be quantitated with an accuracy (RE) within ±20% and a precision (RSD) required to be less than 20%.

#### 3.7.3. Precision and Accuracy

To verify the intra- and inter-day accuracy and precision, three levels of QC samples (LQC, MQC and HQC) and LLOQ in six replicates were analyzed on the same day and on three consecutive days, respectively. The precision was expressed as the RSD% of the measured concentration should be ≤15% and the accuracy was assessed by the RE (within ±15%). 

#### 3.7.4. Extraction Recovery and Matrix Effect

The extraction efficiency of the fourteen analytes was determined by analyzing six replicates of the plasma samples at their respective LQCs, MQCs and HQCs. The extraction recovery was evaluated by comparing the peak areas of blank matrix samples spiked before and after extraction of three QC samples. The matrix effect was acquired by calculating the mean peak areas of processed plasma samples containing the spiked analytes after extraction with the corresponding standard solution. The extraction recovery and the matrix effect were similarly evaluated for the IS at one concentration. 

#### 3.7.5. Stability Experiments

The stability of the fourteen analytes in beagle dogs investigated by analyzing QC samples of each analyte at three QC levels (LQC, MQC and HQC) and each level contained six replicates. The short-term stability was assessed during storage for 4 h at ambient temperature. The long-term stability was determined after the QC samples had been stored for 2 weeks at −80 °C. The freeze-thaw stability was determined after three freeze at −80 °C and thaw cycles. and post-preparative stability was during storage for 12 h after sample preparation at 4 °C. The room temperature stability for stock solution was determined after storing the sample at 25 °C for 24 h. A series of concentrations of stability testing QC samples was gained by the calibration curve which was established from freshly prepared standard samples.

### 3.8. Application to Pharmacokinetic Study

Concentrations of fourteen alkaloids in beagle dog blood were calculated employing the Excel program (Microsoft Corp., Redmond, WA, USA). Pharmacokinetic parameters were calculated using non-compartmental methods. The elimination rate constant (*K_e_*) was calculated employing the linear regression of the terminal points in a semi-log plot of the plasma concentration against time. The elimination half-life (*t*_1/2_) was calculated using the formula.

*t*_1/2_ = 0.693/*K_e_* (1)

The area under the blood concentration versus time curve up to the last quantifiable time point (AUC_0__→t_) was calculated by the linear trapezoidal rule. The area under the plasma concentration –time curve to time infinity (AUC_0→∞_) was calculated as follows:

AUC_0__→__∞_ = AUC_0__→__t_ + C_t_/*K_e_* (2)

The maximum observed blood concentration (*C*_max_) and the time to reach maximum observed blood concentration (*T*_max_) were obtained directly from the concentration-time curve.

## 4. Conclusions

In this study, the UHPLC-MS/MS assay method established in this research for simultaneous determination of A–N provided adequate recovery and matrix effect with good precision and accuracy. According to our knowledge, this is the first report on the pharmacokinetic profile of fourteen alkaloids of *C. bungeana* extract in beagle dogs following administration of a single oral dose. It is expected that these pharmacokinetic results would be contribute to the further research on the action mechanisms and pharmacodynamics study of *C. bungeana*.

## Figures and Tables

**Table 1:** The regression equations, linear ranges and LLOQs for the determination of the analytes in beagle’s plasma.

Compounds	Linear Range (ng/mL)	Regression Equation	R^2^	LLOQ (ng/mL)
Z23	5.34–2670	*Y* = 6.19X + 5.50 × 10^−3^	0.9989	5.34
Coptisine	5.00–2500	*Y* = 9.86X − 7.69 × 10^−3^	0.9985	5.00
Berberrubine	5.53–2763	*Y* = 23.48X − 3.73 × 10^−2^	0.9978	5.53
Sanguinarine	5.00–2500	*Y* = 15.22X − 2.64 × 10^−2^	0.9949	5.00
Worenine	5.55–2775	*Y* = 0.776X + 3.39 × 10^−4^	0.9905	5.55
Berberine	5.40–2700	*Y* = 9.75X + 6.15 × 10^−2^	0.9942	5.40
Jateorhizine	5.00–2500	*Y* = 1.12X + 8.51 × 10^−3^	0.9910	5.00
Columbamine	5.58–2788	*Y* = 0.323X + 6.27 × 10^−3^	0.9918	5.58
Palmatine	5.65–2825	*Y* = 2.238X + 3.34 × 10^−2^	0.9985	5.65
Protopine	4.87–2435	*Y* = 26.65X − 8.12 × 10^−3^	0.9909	4.87
Tetrahydropalmatine	5.07–2535	*Y* = 58.74X − 4.69 × 10^−2^	0.9981	5.07
Corynoline	5.11–2555	*Y* = 75.78X + 1.44 × 10^−2^	0.9904	5.11
8-oxocorynoline	5.80–2900	*Y* = 4.49X − 7.19 × 10^−3^	0.9922	5.80
Acetylcorynoline	6.50–3250	*Y* = 15.10X − 2.09 × 10^−3^	0.9989	6.50

**Table 2:** Precision and accuracy of the determination of fourteen alkaloids in beagle plasma (*n* = 18, 6 replicates per day for 3 days).

Compounds	Spiked Cone. (ng/mL)	Measured Cone. (ng/mL)	Accuracy (%)	Intra-Day Precision (%)	Inter-Day Precision (%)
Z23	5.34	6.24 ± 0.75	13.83	12.15	11.23
	10.86	12.09 ± 1.59	11.37	13.36	11.25
	133.5	152.6 ± 14.59	14.40	9.90	6.20
	2136	1934 ± 120.3	−9.40	5.50	10.10
Coptisine	5.00	4.47 ± 0.61	−10.70	13.90	12.60
	10.00	10.36 ± 1.31	3.58	12.58	12.86
	125.0	139.1 ± 9.12	11.24	6.22	8.68
	2000	1826 ± 109.0	−8.70	6.00	5.70
Berberrubine	5.53	6.26 ± 0.68	13.40	11.20	8.60
	11.05	12.62 ± 1.42	14.21	11.75	6.65
	138.1	130.5 ± 13.62	−5.50	10.80	6.70
	2210	2046 ± 201.5	−7.40	10.30	6.00
Sanguinarine	5.00	5.64 ± 0.79	12.80	14.70	6.50
	10.00	11.32 ± 0.90	13.25	8.42	2.12
	125.0	112.4 ± 7.98	−10.10	6.40	11.10
	2000	2117 ± 218.4	5.87	10.74	6.25
Worenine	5.55	6.17 ± 0.61	11.10	10.30	6.00
	11.10	12.09 ± 0.95	8.90	7.20	12.00
	138.8	128.6 ± 10.19	−7.30	8.00	7.00
	2220	2443 ± 200.7	10.10	8.10	8.70
Berberine	5.40	5.87 ± 0.68	8.70	12.10	6.80
	10.80	11.55 ± 1.43	6.97	12.30	12.64
	135.0	151.3 ± 13.79	12.10	9.30	7.80
	2160	2275 ± 273.5	5.31	11.93	12.74
Jateorhizine	5.00	5.69 ± 0.61	13.90	11.00	8.90
	10.00	11.05 ± 0.88	10.48	7.95	8.15
	125.0	130.1 ± 9.93	4.10	7.96	4.36
	2000	2157 ± 212.1	7.82	9.86	9.66
Columbamine	5.58	5.99 ± 0.73	7.50	12.20	11.90
	11.15	12.48 ± 1.29	11.95	9.98	12.85
	139.4	124.4 ± 12.37	−10.70	9.50	12.70
	2230	2292 ± 219.6	2.78	9.76	8.11
Palmatine	5.65	6.40 ± 0.87	13.40	14.00	9.90
	11.30	12.27 ± 1.01	8.56	8.61	3.90
	141.3	146.1 ± 10.79	3.42	7.73	3.90
	2260	2047 ± 235.1	−9.40	11.50	11.00
Protopine	4.87	5.53 ± 0.61	13.50	11.40	8.60
	9.74	9.89 ± 0.72	1.50	6.40	11.93
	121.8	124.3 ± 5.19	2.11	3.78	6.38
	1948	2216 ± 154.3	13.77	7.36	2.55
Terahydro-palmatine	5.07	5.71 ± 0.57	12.70	10.20	8.40
	10.14	11.41 ± 1.27	12.50	11.44	8.79
	126.8	132.1 ± 8.62	4.19	6.36	7.66
	2028	2228 ± 236.0	9.87	11.15	4.65
Corynoline	5.11	5.73 ± 0.61	12.10	11.10	6.70
	10.22	11.11 ± 1.03	8.69	9.14	9.88
	127.8	139.5 ± 10.59	9.19	7.52	8.11
	2044	2172 ± 241.3	6.28	11.68	5.14
8-oxocorynoline	5.80	4.86 ± 0.67	−12.60	14.00	10.70
	11.60	12.09 ± 1.59	4.27	13.36	11.25
	145.0	125.1 ± 6.39	−13.70	5.20	4.40
	2320	2109 ± 123.1	−9.10	6.20	2.10
Acetylcorynoline	6.50	5.79 ± 0.68	−10.90	11.90	10.30
	13.00	13.78 ± 1.36	6.02	10.12	7.97
	162.5	142.7 ± 15.79	−12.20	11.40	8.20
	2600	2819 ± 208.7	8.40	7.60	5.30

**Table 3:** Matrix effects and extraction recovery for the analytes in beagle plasma (*n* = 6).

Compounds	Spiked Conc. (ng/L)	Matrix Effect (%)	RSD (%)	Recovery (%)	RSD (%)
Z23	10.86	96.98	13.59	80.05	10.35
	133.5	100.02	5.54	87.74	6.82
	2136	100.71	11.52	84.44	5.38
Coptisine	10	99.68	10.66	78.35	10.55
	125	98.35	8.45	86.10	13.60
	2000	99.42	11.29	79.21	6.19
Berberrubine	11.05	95.07	8.25	85.44	8.39
	138.1	102.76	7.14	79.63	9.79
	2210	100.33	5.55	83.76	6.94
Sanguinarine	10	94.08	6.54	82.98	8.14
	125	101.70	2.06	83.94	5.42
	2000	100.59	6.05	87.79	6.73
Worenine	11.10	101.41	8.90	78.67	5.75
	138.8	100.15	3.94	89.67	3.76
	2220	101.77	7.91	88.63	2.80
Berberine	10.80	99.06	9.17	83.24	10.11
	135	100.15	2.81	88.11	4.01
	2160	101.60	7.92	88.36	3.78
Jateorhizing	10	101.01	11.80	82.65	7.11
	125	99.92	4.74	86.10	4.97
	2000	99.66	5.48	87.46	4.28
Columbamine	11.15	100.14	6.80	88.90	7.52
	139.4	100.45	4.11	87.79	6.51
	2230	99.34	5.58	88.75	3.60
Palmatine	11.30	101.91	7.90	82.17	6.47
	141.3	101.49	7.40	86.68	5.99
	2260	99.51	4.11	88.70	4.69
Protopine	9.74	99.78	8.37	78.40	10.49
	121.8	100.86	4.78	89.59	2.22
	1948	99.08	9.96	82.63	7.96
Teahydropalmatine	10.14	99.57	10.28	81.09	8.38
	126.8	101.87	2.68	88.41	3.88
	2028	101.64	7.18	81.94	7.45
Corynoline	10.22	99.31	5.29	84.75	5.77
	127.8	101.63	7.06	84.52	6.18
	2044	101.41	8.70	83.65	10.00
8-oxocorynoline	11.60	100.57	8.74	78.03	9.25
	145	100.92	7.17	87.53	11.00
	2320	99.80	2.36	87.12	5.51
Acetylcorynoline	13	101.50	12.01	78.21	9.53
	162.5	99.72	5.06	84.63	5.88
	2600	101.49	4.77	86.13	5.03
I.S.	2000	99.12	11.88	84.71	8.97

**Table 4:** Stabilities of the analytes in beagle plasma (*n* = 6).

Compounds	Spiked Conc. (ng/mL)	Stability (% RE)				
		Freeze-Thaw	Short-Term	Long-Term	Post-Preparative	Room Temperature for Stock-Solution
Z23	10.86	5.35	10.52	−8.16	12.10	3.65
	133.5	12.92	9.94	9.86	11.32	1.64
	2136	12.43	10.85	9.00	−6.09	2.30
Coptisine	10	10.21	10.66	5.48	7.64	−4.12
	125	8.93	−3.05	6.73	8.40	1.35
	2000	12.83	7.57	13.69	10.58	3.70
Berberrubine	11.05	12.88	11.76	11.85	5.99	−2.05
	138.1	5.15	5.82	3.75	7.97	1.25
	2210	10.06	10.78	11.01	5.08	3.65
Sanguinarine	10	11.45	12.41	13.35	7.77	2.48
	125	8.78	−5.50	7.46	−2.41	3.20
	2000	12.22	9.29	11.08	10.40	−4.02
Worenine	11.10	11.96	−5.32	−6.79	8.10	2.10
	138.8	8.93	3.89	7.99	9.22	−3.48
	2220	11.16	10.03	11.40	13.58	2.59
Berberine	10.80	12.57	9.61	7.75	11.84	2.22
	135	11.60	7.42	7.72	9.39	4.08
	2160	7.71	11.96	7.00	10.36	−3.26
Jateorhizine	10	10.65	7.31	12.90	6.63	1.16
	125	8.68	3.05	10.30	8.51	−3.58
	2000	6.24	6.64	8.26	8.41	1.32
Columbamine	11.15	11.50	8.47	9.66	8.80	4.02
	139.4	12.73	4.02	11.77	5.50	1.62
	2230	5.60	9.67	10.24	12.44	2.50
Palmatine	11.30	10.26	9.42	7.79	6.92	2.77
	141.3	8.08	−6.30	10.77	9.22	−4.66
	2260	8.37	6.73	11.04	−7.08	1.30
Protopine	9.74	3.72	5.52	−5.39	1.65	2.17
	121.8	4.17	8.75	7.93	11.35	−3.87
	1948	10.26	12.18	13.11	10.57	2.02
Terahydropalmatine	10.14	11.34	9.24	−5.67	10.82	2.97
	126.8	12.23	3.11	11.88	8.29	4.65
	2028	10.09	5.15	10.23	8.76	1.28
Corynoline	10.22	6.11	−2.63	13.95	7.14	4.42
	127.8	6.78	9.25	6.81	−2.98	−3.74
	2044	7.95	6.81	9.74	5.40	1.99
8-oxocorynoline	11.60	1.18	1.62	3.08	3.71	−4.08
	145	9.69	−3.92	5.35	10.01	3.77
	2320	−3.58	−2.89	3.13	10.60	2.30
Acetylcorynoline	13	5.17	6.47	8.62	12.65	−1.83
	162.5	−3.75	6.74	7.56	−3.44	2.57
	2600	−4.20	−4.81	7.32	2.05	3.69

**Table 5:** Pharmacokinetic parameters of the fourteen constituents in beagles after oral administration of *C. bugeana* (mean ± SD, *n* = 6).

Analytes	*C*_max_ (ng/mL)	*t*_max_ (h)	*t*_1/2_ (h)	AUC_0__→t_ (ng h/mL)	AUC_0__→∞_ (ng h/mL)
Z23	237.0 ± 13.66	0.96 ± 0.10	6.34 ± 1.21	682.2 ± 74.81	746.7 ± 95.24
Coptisine	2256 ± 255.9	0.92 ± 0.13	2.48 ± 0.33	5957 ± 204.6	6032 ± 194.1
Berberrubine	450.1 ± 61.61	0.96 ± 0.10	4.88 ± 1.72	1156 ± 94.24	1204 ± 86.29
Sanguinarine	533.4 ± 29.12	0.96 ± 0.10	5.63 ± 1.36	1260 ± 107.4	1346 ± 109.4
Worenine	123.4 ± 10.34	0.71 ± 0.10	5.95 ± 0.84	396.9 ± 42.21	482.2 ± 91.51
Berberine	1369 ± 312.7	0.92 ± 0.13	2.49 ± 0.14	3691 ± 141.0	3767 ± 139.0
Jateorhizine	74.16 ± 8.71	0.54 ± 0.10	6.29 ± 1.09	151.1 ± 6.42	163.2 ± 11.04
Columbamine	2130 ± 195.7	0.92 ± 0.13	2.87 ± 0.42	6826 ± 170.0	7026 ± 289.6
Palmatine	1902 ± 84.86	0.96 ± 0.10	8.56 ± 1.29	8037 ± 459.3	9582 ± 596.2
Protopine	433.7 ± 73.92	0.88 ± 0.13	4.92 ± 1.49	1029 ± 106.2	1079 ± 135.6
Terahydropalmatine	205.0 ± 22.39	0.92 ± 0.13	5.58 ± 1.46	584.7 ± 59.85	622.5 ± 52.46
Corynoline	223.9 ± 15.29	0.96 ± 0.10	4.25 ± 0.81	733.8 ± 57.45	755.3 ± 58.92
8-oxocorynoine	94.49 ± 22.77	0.92 ± 0.13	6.98 ± 1.76	260.5 ± 64.21	283.6 ± 62.41
Acetylcorynoline	106.7 ± 4.63	0.58 ± 0.13	5.52 ± 0.93	229.7 ± 18.60	244.4 ± 26.16
